# Identification of miRNA-mRNA-TF regulatory networks in peripheral blood mononuclear cells of type 1 diabetes

**DOI:** 10.1186/s12902-022-01038-y

**Published:** 2022-05-09

**Authors:** Wanqiu Wang, Huan Wang, Yuhong Liu, Liu Yang

**Affiliations:** 1 Department of Endocrinology and Metabolism, The First Affiliated Hospital, Shihezi University School of Medicine, Shihezi, Xinjiang China; 2 Department of Clinical Laboratory, Shandong Cancer Hospital and Institute, Shandong First Medical University and Shandong Academy of Medical Sciences, Jinan, Shandong China

**Keywords:** Type 1 diabetes (T1D), MicroRNAs, Differentially expressed genes (DEGs), Transcription factors (TFs), Regulatory networks

## Abstract

**Background:**

Type 1 diabetes (T1D) is a T lymphocyte-mediated and B lymphocyte-assisted autoimmune disease. We aimed to identify abnormally expressed genes in peripheral blood mononuclear cells (PBMCs) of T1D and explore their possible molecular regulatory network.

**Methods:**

Expression datasets were downloaded from the Gene Expression Omnibus (GEO) database. Then, the differentially expressed genes (DEGs) and differentially expressed miRNAs (DEmiRNAs) were identified, and functional enrichment and immune cell infiltration analysis were performed. The starBase, miRTarBase, TarBase, JASPAR, ENCODE, and TRRUST databases constructed the miRNA-mRNA-TF regulatory network. The ROC curves were plotted to evaluate the sensitivity and specificity of miRNAs and mRNAs.

**Result:**

A total of 216 DEGs directly or indirectly related to type I diabetes mellitus, natural killer cell-mediated cytotoxicity, Th1, and Th2 cell differentiation, and the IL-17 and *TNF* signaling pathways were obtained. The miRNA-mRNA-TF network indicates that miR-320a and *SOX5* are the only miRNAs and TFs that both target *ADM* and *RRAGD*. The ROC curves showed that *ADM* (0.9375), *RRAGD* (0.8958), and hsa-mir-320a (0.9417) had high accuracy in T1D diagnosis.

**Conclusion:**

The constructed regulatory networks, including miR-320a/*ADM/SOX5* and miR-320a/*RRAGD/SOX5,* may provide new insight into the mechanisms of development and progression in T1D.

**Supplementary Information:**

The online version contains supplementary material available at 10.1186/s12902-022-01038-y.

## Introduction

Diabetes is a chronic disease. Compared with type 2 diabetes (T2D), type 1 diabetes (T1D) is an autoimmune-mediated disease in which selective destruction of pancreatic islet β cells is caused by multiple complex crosstalks of genetic and environmental determinants [1]. Emerging evidence indicates that miRNA, as a key regulator of β-cell physiology, is essential for fine-tuning gene expression regulation in the differentiation of insulin-producing cells and contributing to the acquisition and management of unique phenotypes in diabetes [2]. Namely, dysregulation of miRNA expression contributes to β-cell dysfunction and facilitates the development of multiple forms of diabetes mellitus.

As one of the critical biological players, transcription factors (TFs) are crucial for the pathophysiological process of multiple diseases, including cancer, autoimmunity, and diabetes [3]. Given that the therapeutic regulation of TFs by drugs may affect significant changes in gene expression patterns and could improve disease conditions, identifying the key TFs in the pathophysiology of T1D may promise better antidiabetic drugs. In addition, early research has shown that disorders of components in peripheral blood mononuclear cells (PBMCs), including T cells and B cells, and any factors that can affect their functional balance may be involved in the occurrence and development of T1D [4].

Although the importance of miRNAs in diabetes has been slowly recognized in recent years [2, 5, 6], little is known about the complex molecular regulation mechanisms of miRNAs, mRNAs, and TFs in the development of T1D. Therefore, it is crucial to explore the molecular regulatory mechanisms of miRNAs, mRNAs, and TFs in the PBMCs of patients with T1D, which may be valuable for diagnosis and treatment.

## Materials and methods

### Data source

The expression datasets of GSE55098 [4], GSE55099, and GSE33440 [7] were downloaded from the Gene Expression Omnibus (GEO) database (website: http://www.ncbi.nlm.nih.gov/geo/) [8]. The three datasets contain a total of 40 newly diagnosed T1D patients and 26 normal controls. The details of GSE55098, GSE55099, and GSE33440 are listed in Table 1. In this study, the GSE55098 dataset was utilized for DEG analysis, functional enrichment analysis, ssGSEA, and receiver operating characteristic (ROC) curve analysis; the GSE55099 dataset was mainly used for differential microRNA expression analysis. The DEGs of GSE33440 were utilized only to take the intersection with the DEGs of GSE55099 to obtain the hub genes.

**Table 1:** Details of the expression data GSE55098, GSE55099, and GSE33440 in the GEO database. N, normal controls; P, patients with type 1 diabetes mellitus; PBMCs, peripheral blood mononuclear cells

GEO accession	Platform	Samples (N:P)	Type
GSE55098	GPL570 ( [HG-U133_Plus_2] Affymetrix Human Genome U133 Plus 2.0 Array)	10:12	PBMC
GSE55099	GPL8786 ( [miRNA-1] Affymetrix Multispecies miRNA-1 Array)	10:12	PBMC
GSE33440	GPL6947 ( Illumina HumanHT-12 V3.0 expression beadchip)	6:16	PBMC

### Data preprocessing

Based on the annotation file of the platform, the corresponding genes with the probes were further matched, and probes that did not map to genes were removed. The expression of the same gene in multiple probes is expressed as the mean value. The differentially expressed genes (DEGs) and differentially expressed miRNAs (DEmiRNAs) between the peripheral blood mononuclear cells of T1D and non-T1D patients under the criteria of |log fold change|> 0.5 and *P* < 0.05 were obtained by using the “limma” [9] package.

### Functional enrichment and immune cell infiltration analysis

To further investigate the major biological functions of DEGs, the Gene Ontology (GO) and Kyoto Encyclopedia of Genes and Genomes (KEGG) pathways [10] were explored in GSE55098 based on the "clusterProfiler" [11] package with the criteria of q-value < 0.05. Moreover, the single-sample gene set enrichment analysis (ssGSEA) algorithm in the “GSVA” package [12] was utilized to quantify the relative abundance of 24 immune cell types [13] infiltration within the entire PBMC subset of profile GSE55098.

### Hub gene selected and evaluation

In this study, intersecting genes between GSE33440 and GSE55098 were defined as hub genes. Then, we utilized Pearson correlation and Wilcoxon rank-sum tests to assess the association of immune cell infiltration with the expression of these hub genes. Moreover, receiver operating characteristic (ROC) curves and related area under the curve (AUC) values were performed to evaluate the sensitivity and specificity of hub genes and miRNAs for T1D diagnosis. The STATS package calculated the correlation between seven hub genes.

### Construction of the miRNA-mRNA-TF target regulatory network

Based on the current miRNA-mRNA interaction regulatory network prediction database, starBase (Version 2.0), miRTarBase (Version 8.0), and TarBase (Version 8.0) were used to predict the potential upstream regulatory miRNAs of hub genes [14–16]. Moreover, the JASPAR, ENCODE, and TRRUST [17–19] databases were utilized to predict the possible transcription factors of seven hub genes. The intersecting TFs that appeared in two transcription factor prediction databases were selected as the targeted TFs, and the targeted miRNAs were obtained in the same way. Based on starBase, miRTarBase, TarBase, JASPAR, ENCODE, TRRUST, and DEmiRNAs, we further explored the effect of the miRNAs and transcription factors (TFs) that targeted the hub genes related to T1D. Visualization of the miRNA-mRNA-TF target regulatory network was performed by Cytoscape software [20].

## Result

### Identification of DEGs

**Fig. 1:**
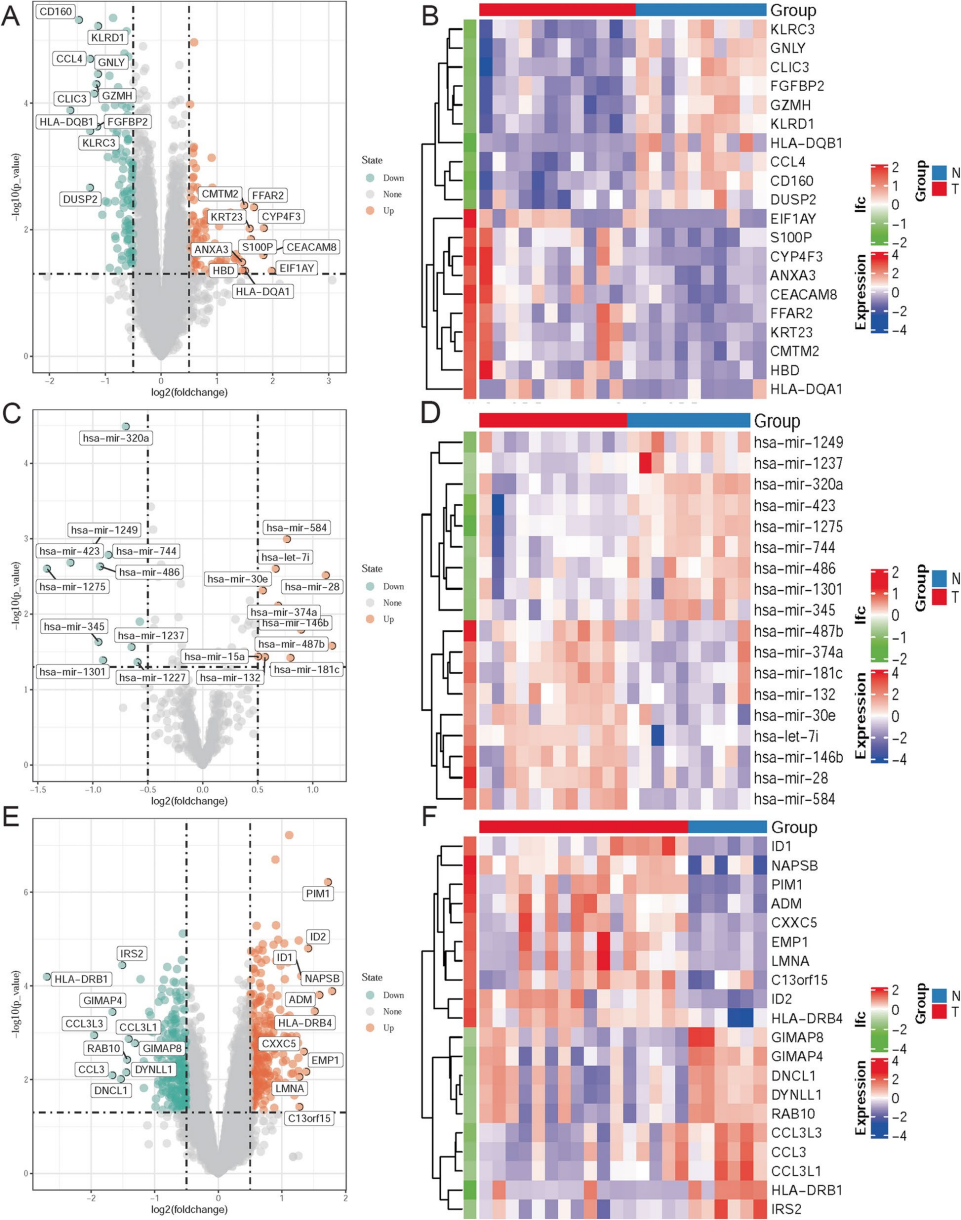
The volcano map and heatmap. **A**, **B** The top 10 up- and downregulated DEGs in GSE55098. **C**, **D** The top 10 up- and downregulated DEmiRNAs in GSE55099. **E**, **F** The top 10 up- and downregulated DEGs in GSE33440

Based on the criteria of |log FC|> 0.5 and *P* < 0.05 of the data preprocessing specified in the Materials and Methods section, we identified 21 DEmiRNAs (10 up- and 11 downregulated miRNAs), 216 DEGs (99 up- and 117 downregulated genes) and 545 DEGs (296 up- and 249 downregulated genes) in GSE55098, GSE55099 and GSE33440, respectively (Supplementary Table 1). Moreover, we visualized the top 10 up-and downregulated DEmiRNAs and DEGs in related datasets by volcano mapping and heat mapping (Fig. 1).

### Functional enrichment and immune cells infiltration

**Fig. 2:**
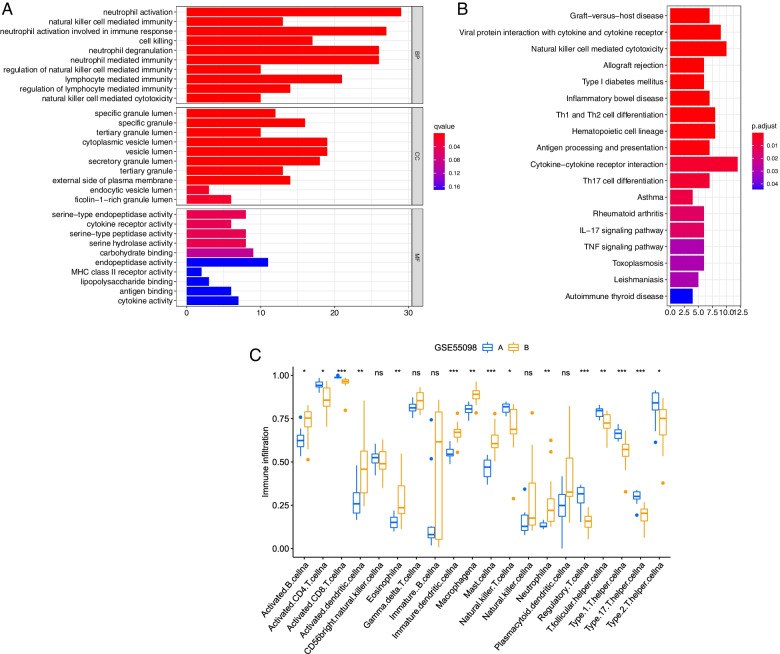
Immune cell infiltration and functional enrichment analysis. **A** The expression of multiple immune cell types between the T1D and no-T1D groups in GSE55098. **B** Bar graph of GO enrichment analysis. **C** Bar graph of KEGG pathway enrichment analysis. GO Gene Ontology, KEGG Kyoto Encyclopedia of Genes and Genomes. "N" and "T" means No-T1D and T1D patients respectively

To further explore the functional implications, we performed GO and KEGG functional enrichment analysis of DEGs in the GSE55098 dataset (Supplementary Table 2). The GO results indicated that these DEGs were mainly associated with neutrophil activation involved in the immune response, natural killer cell-mediated immunity regulation, and lymphocyte-mediated immunity in the biological process analysis (Fig. 2B). KEGG results showed that these DEGs were directly or indirectly related to type I diabetes mellitus, natural killer cell-mediated cytotoxicity, Th1, and Th2 cell differentiation, cytokine-cytokine receptor interaction, Th17 cell differentiation, the IL-17 signaling pathway, and the TNF signaling pathway (Fig. 2C). Moreover, we noticed that there was a significant difference in the expression of multiple immune cell types between the T1D and no-T1D groups (Fig. 2A), including activated CD8 T cells, immature dendritic cells, mast cells, regulatory T cells, type 1 T helper cells, and type 17 T helper cells (*P* < 0.001).

### Hub gene evaluation

**Fig. 3:**
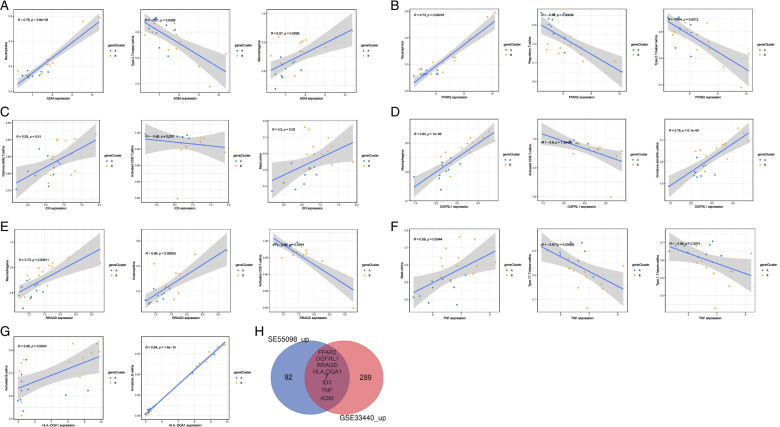
Correlation analysis between immune cell abundance and hub genes. **A** *ADM*. **B** *FFAR2.* **C** *ID3*. **D** *OGFRL1*. **E** *RRAGD.* **F** *TNF.* **G** *HLA-DQA1*. **H** Venn diagram of seven hub genes in the GSE33440 and GSE55098 datasets

**Fig. 4:**
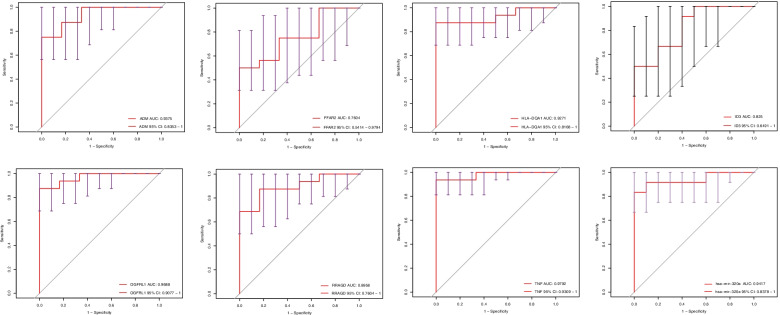
The receiver operating characteristic (ROC) curve and related area under the curve (AUC) value of seven hub genes and hsa-miR-320a

By taking the intersecting genes of the DEGs in GSE33440 and GSE55098 as the target genes of this study, we finally obtained seven upregulated DEGs as hub genes, including *ADM*, *FFAR2*, *HLA-DQA1*, *ID3*, *OGFRL1*, *RRAGD*, and *TNF* (Fig. 3H). ssGSEA is a deconvolution algorithm that assesses the level of immune cell infiltration in samples based on the expression level of immune cell-specific marker genes [10]. Based on this algorithm, we found that these hub genes are mainly related to the expression of multiple immune cell types, including neutrophils, type 2 T helper cells, macrophages, regulatory T cells, gamma delta T cells, activated CD8 T cells, mast cells, immature dendritic cells, eosinophils and type 17 T helper cells (Fig. 3A-G). ROC curves and AUC values were plotted to evaluate the sensitivity and specificity of hub genes for T1D diagnosis. The results indicated that these hub genes were highly accurate in T1D diagnosis (Fig. 4). The AUCs were 0.9375 (*ADM*), 0.9792 (*TNF*), 0.9688 (*OGFRL1*), 0.9271 (*HLA-DQA1*), 0.8958 (*RRAGD*), 0.8250 (*ID3*), and 0.7604 (*FFAR2*). Similarly, miR-320a also has high sensitivity and specificity for T1D diagnosis, and the AUC was 0.9417 (Fig. 4H).

### miRNA—mRNA-TF target regulatory network

**Fig. 5:**
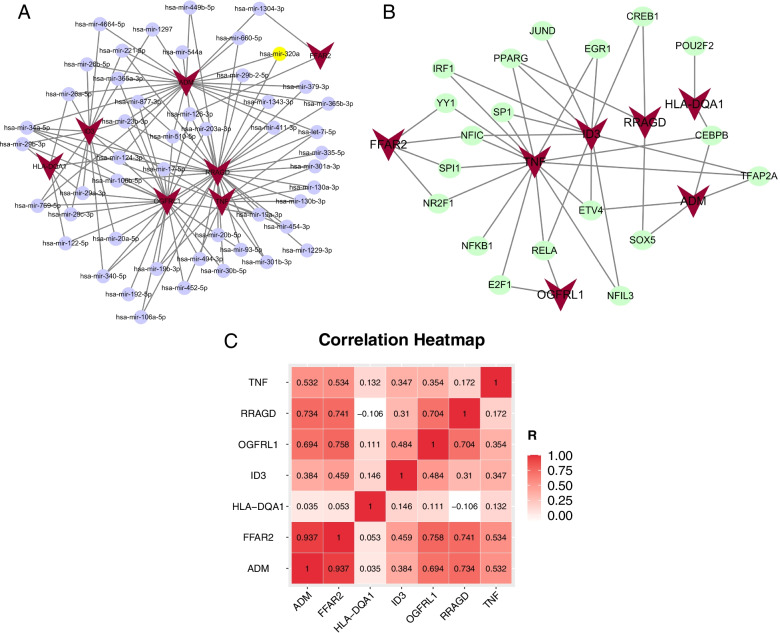
The predicted miRNA-mRNA-TF target regulatory network. **A** The miRNA-mRNA target regulatory network. **B** The TF-mRNA target regulatory network. **C** The correlation heatmap of seven hub gens

To further explore the regulatory relationship in hub genes, TFs, and miRNAs, we constructed a miRNA-mRNA-TF target regulatory network in this study and then visualized the network with Cytoscape software. Among them, 25 miRNAs targeted *ADM*, 33 miRNAs targeted *RRAGD*, 26 miRNAs targeted *OGFRL1*, 15 miRNAs targeted *ID3*, 2 miRNAs targeted *FFAR2*, and 6 miRNAs targeted *HLA-DQA2*, and 12 miRNAs targeted *TNF*. miR-320a, as a DEmiRNA, appeared in the miRNA prediction database (Fig. 5A; Supplement Table 3). A total of 27 TFs targeted seven hub genes in the TRRUST database, 66 TFs in the JASPAR database, and 139 TFs in the ENCODE database; among them, 19 TFs appeared in more than two databases (Fig. 5B; Supplement Table 4). Remarkably, miR-320a and *SOX5* are the only miRNAs and TFs targeting *ADM* and *RRAGD.* Moreover, there was a high correlation between the *ADM* and *RRAGD* (Fig. 5C).

## Discussion

This study analyzed a PBMC microarray dataset involving T1D patients and normal controls and obtained 216 DEGs. The functional enrichment analysis results indicate that these DEGs are closely related to various immunity regulation pathways and biological processes. Since T1D is a T lymphocyte-mediated and B lymphocyte-assisted autoimmune disease [21], it is not surprising that these DEGs are closely associated with immune processes. Then, we obtained seven hub genes: *ADM, FFAR2, HLA-DQA1, ID3, OGFRL1, RRAGD*, and *TNF*. These hub genes are not only associated with the expression of multiple immune cell types but also have a high accuracy in T1D diagnosis, with AUCs of 0.9375 (*ADM*), 0.9792 (*TNF*), 0.9688 (*OGFRL1*), 0.9271 (*HLA-DQA1*), 0.8958 (*RRAGD*), 0.8250 (*ID3*), and 0.7604 (*FFAR2*). Finally, based on the miRNA prediction databases and transcription factor prediction databases, we constructed a miRNA-mRNA-TF target regulatory network that included 49 miRNAs, 7 mRNAs, and 19 TFs. Remarkably, miR-320a and *SOX5* are the only miRNAs and TFs targeting *ADM* and *RRAGD*.

Type 1 diabetes (T1D) is a complex autoimmune disease in which the development of pathology requires the crosstalk between various immune cells [22]. As one of the essential innate immune cells, Neutrophils have been required for initiating diabetogenic T cell responses in non-obese diabetic (NOD) mice [23]. Neutrophils infiltrated the pancreas and could form neutrophil extracellular traps that induce dendritic cell activation leading to Th1 polarization of T1D patients [24] and escalating the activating macrophages-related inflammation through modulating inflammatory cytokines directly or indirectly [25]. Interestingly, Macrophages can stimulate β-cells by secreting IL-1β to produce CXCR2 ligands, which are contributed to neutrophil recruitment in pancreatic islets [26]. However, some questions remain: Are there some trinsic mechanisms that can different immune subsets recruiting (such as Neutrophils and Macrophages), affect their pathogenic properties, prolong and enhance their inflammatory activity, and perhaps promote β-cell destruction?

This study found that *ADM* and *RRAGD* in the regulatory network are closely associated with Macrophages and/or Neutrophils (*P* < 0.01). Early studies reported that *ADM* is elevated in the plasma of patients with pancreatic dysfunctions, including T1D [27]. Zudaire et al. [28] further indicated that elevated *ADM* increases circulating glucose levels, while a blocking monoclonal antibody of *ADM* has the opposite effect and ameliorates postprandial recovery. Given the localization of *ADM* in the pancreas and experimental evidence, we hypothesize that *ADM* may play an important role in maintaining insulin homeostasis and normoglycemia and altering pancreatic physiologies. Although no evidence directly indicates that *ADM* mediates pancreatic β-cell damage by macrophages, *ADM* production is related to increased expression of monocyte/macrophage differentiation [29]. The study by Xu et al. shows that *ADM* can enhance the migration and invasion of myelomonocytic cells by activating multiple signaling pathways, including *MAPK*, *PI3K/Akt,* and eNOS in pancreatic cancer, as well as promote myelomonocytic cells trans-endothelial migration through increased expression of *VCAM-1* and *ICAM-1*. Conversely, knockout or antagonist of *ADM* significantly inhibited monocyte recruitment [30]. As for neutrophils, *ADM* can exert pro-inflammatory effects in the cutaneous microvasculature to mediate vasodilation and promote edema formation related to acute inflammatory processes, thereby enhancing neutrophil accumulation in the skin microvasculature [31]. Another hub gene, *RRAGD,* is a monomeric guanine nucleotide-binding protein that can act as a molecular switch to interact with the C terminal region of leucyl tRNA synthase, which acts as a direct sensor of the amino acid leucine and is contributed to the activation of *mTORC1* [32]. Studies have shown that *mTORC1* is closely related to glucose metabolism, lipid metabolism, and islet resistance [33]. In the state of excess nutrients, the activation of mTORC1 signaling may promote oxidative stress, ER stress, and inflammation, thereby damaging pancreatic β cells [32]. The activation of mTORC1 signaling may also impair autophagic flux, consequently triggering the death of pancreatic β-cells [34] and leading to T2D pancreatic islet failure or T1D. Namely, *RRAGD* may participate in the occurrence and development of diabetes by activating *mTOR1*. Moreover, activated *mTORC1* regulates inflammatory immune responses in various innate immune cells ( such as monocytes, macrophages, and dendritic cells) in the [35]. Manipulation of genes in its pathway has been shown to alter macrophage polarization and the production of inflammatory and immunoregulatory cytokines in mice [36]. Specifically, *mTORC1* inhibits apoptosis and induces macrophage proliferation by inducing the expression and metabolic reprogramming of the cell cycle kinase *CDK4* [37].

In further investigation of regulatory mechanisms of hub genes in T1D, we found that miR-320a and *SOX5* are the only miRNA and TF that both target *ADM* and *RRAGD*. Knowledgeably, abnormal miRNA expression can cause some pathological conditions. Previous research indicated that miR-320 is lowly expressed in the serum of people with diabetes [38], especially those with diabetic retinopathy [39]. Similarly, decreased expression of miR-320 was observed in the kidneys of glucose-induced diabetic rats and human umbilical vein endothelial cells [40]. Notably, miR-320a is significantly downregulated in the human islets of T2D subjects, which is accompanied by an increase in glucagon, and the increased glucagon as a marker of diabetes will worsen hyperglycemia [41]. Moreover, the downregulation of miR-320a participates in regulating multiple signaling pathways, including *PI3K/AKT* and *MAPK/ERK*, by targeting *IGF-1R*, which is associated with obesity and T2D [42]. However, there are inconsistencies in the results of some studies both on miR-320a and diabetes. Ling et al. revealed that miR-320 is upregulated in insulin-resistant adipocytes, and anti-miR-320 oligonucleotides can reverse insulin resistance and increase adipocyte insulin sensitivity by insulin *PI3K* signaling pathways [43]. Given the inconsistent role of miR-320a in diabetes, a future longitudinal study of miR-320 and diabetes-related progression is needed to understand the specific expression and molecular regulatory mechanisms.

*SOX5* is a member of the SOX transcription factor family that participates in embryonic development and cell fate regulation and is expressed in both the nucleus and cytoplasm of human α- cells and β- cells [44]. Cytosolic expression of *SOX5* was especially evident in α-cells; conversely, nuclear *SOX5* was reduced by 67% in T2D β-cells compared with nondiabetic β-cells. Moreover, Axelsson et al. further indicated that knockdown of *SOX5* attenuates glucose-stimulated insulin secretion and reduces the expression of L-type Ca2 + channels. In contrast, overexpressed *SOX5* can restore impaired glucose-stimulated insulin secretion and increase insulin secretion by 18% in T2D islets [44].

Although understanding the regulatory network of miRNAs, TFs, and mRNA is crucial for revealing the underlying mechanisms of T1D and identifying and constructing a molecular regulatory network, some limitations remain in this study. This study is only based on the GEO database to conduct preliminary exploration at the level of bioinformatics, and further comprehensive experiments in vitro and in vivo are needed to verify these constructed regulatory mechanisms. Therefore, obtaining insight into the gene interactions and molecular mechanisms of diabetes still has a long way to go.

## Conclusions

Generally, the constructed regulatory mechanism networks, including mir-320a/*ADM/SOX5* and mir-320a/*RRAGD/SOX5*, are robust and promising and may provide new insight into the mechanisms of development and progression of T1D.

## Supplementary Information

**Additional file 1.**

**Additional file 2.**

**Additional file 3.**

**Additional file 4.**

## Data Availability

The entire sequencing profile data in this study come from the Gene Expression Omnibus (GEO) database (website: http://www.ncbi.nlm.nih.gov/geo/).
